# Genistein up-regulates miR-20a to disrupt spermatogenesis via targeting Limk1

**DOI:** 10.18632/oncotarget.17637

**Published:** 2017-05-05

**Authors:** Hao Gu, Wei Wu, Beilei Yuan, Qiuqin Tang, Dan Guo, Yiqiu Chen, Yankai Xia, Lingqing Hu, Daozhen Chen, Jiahao Sha, Xinru Wang

**Affiliations:** ^1^ State Key Laboratory of Reproductive Medicine, Institute of Toxicology, Nanjing Medical University, Nanjing 211166, China; ^2^ Key Laboratory of Modern Toxicology of Ministry of Education, School of Public Health, Nanjing Medical University, Nanjing 211166, China; ^3^ Department of Central Laboratory, Huai’an First People's Hospital, Nanjing Medical University, Huai’an 223002, China; ^4^ State Key Laboratory of Reproductive Medicine, Wuxi Maternal and Child Health Care Hospital Affiliated to Nanjing Medical University, Wuxi 214002, China; ^5^ State Key Laboratory of Reproductive Medicine, Department of Obstetrics, Obstetrics and Gynecology Hospital Affiliated to Nanjing Medical University, Nanjing 210004, China; ^6^ State Key Laboratory of Reproductive Medicine, Department of Histology and Embryology, Nanjing Medical University, Nanjing 211166, China

**Keywords:** genistein, miR-17-92 cluster, miR-20a, Limk1, spermatogenesis

## Abstract

Genistein (GEN) is one of the isoflavones that has effect on male reproduction. However, the underlying mechanism remains unknown. miRNAs are a type of small non-coding RNAs that play important roles in spermatogenesis. We measured the GEN levels and miR-17-92 cluster expression in infertile subjects and found that miR-17-92 might be involved in GEN induced abnormal spermatogenesis. To clarify, we fed adult ICR mice with different doses of GEN (0, 0.5, 5, 50 and 250 mg/kg/day) for 35 days to study the underlying mechanism. We found that sperm average path velocity, straight-line velocity and eurvilinear velocity of the mice orally with GEN at 5mg/kg/day were significantly decreased, the expression levels of miR-17 and miR-20a in mice testis were higher in corresponding group. We also found miR-20a was the only miRNA that differentially expressed both in human and mice. By applying bioinformatics methods, Limk1 was predicted to be the target gene of miR-20a that is involved in spermatogenesis. Limk1 were significantly decreased in the corresponding group. Dual-luciferase report assay also proved that miR-20a could directly target Limk1. These results implied that Limk1 might be the target gene of miR-20a that is involved in GEN induced abnormal spermatogenesis.

## INTRODUCTION

About 12% of reproductive-aged couples are suffering from infertility [1]. Male infertility is defined as failure to father a child successfully after 12 months or more of regular intercourse [2]. Endocrine disrupting chemicals (EDCs) are some chemicals that may disrupt endogenous hormone to reduce adverse effects on immune response, development and reproduction in mammals [3]. As a class of EDCs, phytoestrogens (PEs) are natural plant-derived non-steroidal compounds [4]. Because of their ability to mimic the structure of oestradiol, they can bind both estrogen receptors (ER-*α* and ER-*β*) [5]. Isoflavones, coumestans, naringin and lignans are four major classes of PEs [6]. Isoflavones such as genistein (GEN) are mainly found in soy and its products [7]. Higher doses of GEN and beta-lapachone could suppress acrosome reaction through cytotoxic effects on sperm cell membrane in rats [8], and our laboratory also found that exposures to GEN were significantly related to idiopathic male infertility [6]. However, the underlying mechanism remains unclear.

microRNAs (miRNAs) are family of 19-24 nucleotides, single-stranded non-coding RNAs that play significant roles in regulating their target genes expression by post-transcriptional gene silencing through base-pair [9]. It is widely accepted that EDCs could play biological function by dysregulating expression of miRNAs [10, 11]. Similarly, GEN could inhibit cancer cell proliferation via changing the expression of miRNAs [12]. But whether GEN could induce abnormal spermatogenesis via miRNAs has not been reported before. The miR-17-92 cluster is one of the best-studied miRNA clusters. This cluster includes miR-17, miR-18a, miR-19a, miR-19b-1, miR-20a, and miR-92a-1 [13, 14]. Many researchers have reported that miR-17-92 cluster is involved in many bioprocesses like carcinogenesis [15, 16], immune response [17], cardiovascular diseases [18], neurodegenerative diseases [19] and so on. As for our previously study, miR-17-92 cluster was over-expressed in idiopathic infertile males with non-obstructive azoospermia (NOA) [20], and other researchers indicated that miR-17-92 was involved in the regulation of spermatogonial differentiation and spermatogenesis in mice [21, 22]. To clarify, the aim of this study was to determine whether and how miR-17-92 cluster plays roles in abnormal spermatogenesis induced by GEN.

## RESULTS

### Characteristics of the study subjects

All 130 subjects were averagely divided into 4 groups according to the urinary GEN concentration. The ranges of the GEN concentration were < 16.02 ng/ml (Group 1), 16.02-76.32 ng/ml (Group 2), 76.32-281.96 ng/ml (Group 3), and > 281.96 ng/ml (Group 4). Group 1 was the lowest level of GEN exposure, so Group 1 was considered to be the control group in this study. There was no significant difference between age, BMI, smoking and drinking between different groups while compared with Group 1. However, the levels of GEN were statistically significantly different from Group 1 (Table 1).

**Table 1:** Characteristics of the study population

Characteristic	Total	Group 1	Group 2	Group 3	Group 4	*P* value
	(n = 130)	(n = 32)	(n = 33)	(n = 33)	(n = 32)	
**Genistein (ng/ml, mean ± SEM)**	236.11 ± 30.39	7.83 ± 0.76	42.58 ± 3.31	163.22 ± 10.38	739.13 ± 64.92	< 0.01
**Age (year, mean ± SEM)**	28.47 ± 0.36	29.31 ± 0.80	28.40 ± 0.72	28.06 ± 0.64	28.12 ± 0.68	0.583
**BMI (kg/m^2^, mean ± SEM)**	23.21 ± 0.30	23.11 ± 0.63	22.79 ± 0.40	23.07 ± 0.61	23.94 ± 0.71	0.567
**Smoking [n (%)]**						
**Yes**	69 (53.1)	16 (50.0)	23 (69.7)	17 (51.5)	14 (43.8)	
**No**	61 (46.9)	16 (50.0)	10 (30.3)	16 (48.5)	18 (56.3)	0.178
**Drinking [n (%)]**						
**Yes**	65 (50.0)	15 (46.9)	14 (42.4)	14 (42.4)	20 (65.6)	
**No**	65 (50.0)	17 (53.1)	19 (57.6)	19 (57.6)	11 (34.4)	0.242

### Relations between GEN and sperm characteristics in human

**Figure 1:**
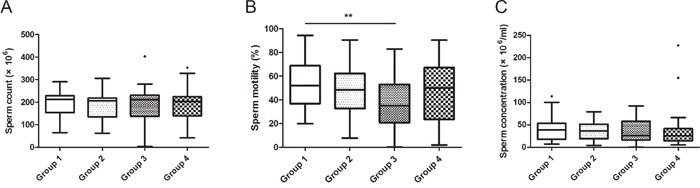
Relationship between GEN exposure and sperm characteristics in recruited subjects All 130 subjects were divided into 4 groups according to their exposure level of GEN. The ranges of GEN in the groups were: Group 1 (< 16.02 ng/ml), Group 2 (16.02-76.32 ng/ml), Group 3 (76.32-281.96 ng/ml), Group 4 (> 281.96 ng/ml). **(A, B and C)** represents sperm count, sperm motility and sperm concentration, respectively. Date was shown as turkey because the exposures of GEN were varied widely. **P < 0.01 versus Group 1.

We compared the subjects’ sperm characteristics in different groups and found that the sperm motility was lower in Group 3 (*P* < 0.01), while the differences of other sperm characteristics didn't reach a statistically significant level (Figure 1, Supplementary Figure 1).

### Relations between GEN concentration and seminal plasma miR-17-92 expressions in human

**Figure 2:**
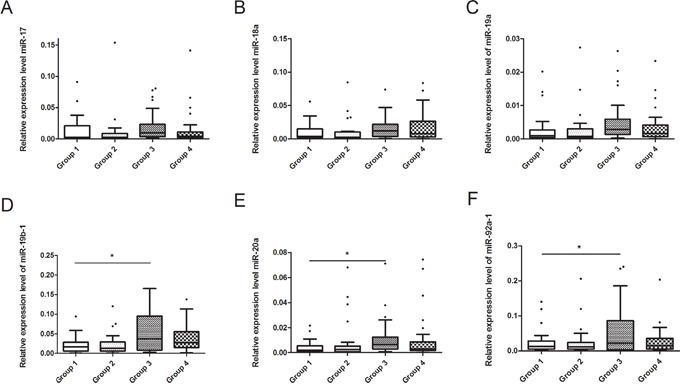
Relationship between GEN and seminal plasma miR-17-92 expressions in recruited subjects **(A-F)** represents miR-17, miR-18a, miR-19a, miR-19b-1, miR-20a and miR-92a-1, respectively. *P < 0.05 versus Group 1.

Seminal plasma samples were obtained from the recruited subjects. We test the expression of U6 in different groups and found that, expression of U6 were not correlated with GEN exposure, suggested that U6 was not regulated by GEN, and it is suitable for reference. It is interesting that the relative expression levels of miR-19b-1, miR-20a and miR-92a-1 were higher in Group 3 compared to Group 1 (Figure 2, *P* < 0.05). The relative expression levels of other 3 miRNAs were nearly equal in four groups (Figure 2).

### Changes in behavior, body weight and organ mass of the mice

The food and water consumption of the mice were similar among the 4 groups. All the mice were weighted daily and there was no statistically significant difference in initial weight and weight changes between different groups (Supplementary Figure 2A). After weighing the testicles of each mouse, we found that there was no obvious difference of testicular index between the control and GEN treated groups (Supplementary Figure 2B).

### Histopathology

We compared detachment (characterized as breaking spermatocytes from seminiferous epithelium), vacuolization (characterized as empty spaces in the seminiferous tubules), and sloughing (characterized as release of clusters of germ cells into the lumen of the seminiferous tubules) in testis of the mice. However, testicular sections from control and treated groups showed no obvious abnormalities in these histopathological alterations (Supplementary Figure 3).

### Changes in sperm quality

To investigate the effects of GEN on sperm quality, sperm concentration, sperm motility, progressive, VAP (average path velocity), VSL (straight-line velocity), VCL (eurvilinear velocity), ALH (amplitude of lateral head displacement), BCF (beat-cross frequency), STR (straightness), LIN (linearity) and sperm activity of the mice were detected respectively. It is interesting that after treatment with 5 mg/kg/day GEN, the values of VAP, VSL and VCL were significantly reduced compared with the control group (Supplementary Figure 4, *P* < 0.05). However, mice treated with GEN at other doses have similar sperm quality compared to the control group (Supplementary Figure 4).

### Changes of serum sex hormone levels

**Figure 3:**
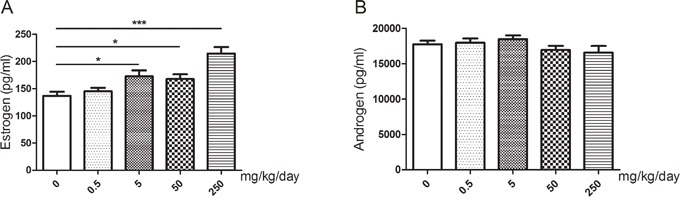
Changes of serum sex hormone levels of mice after treated with GEN at different doses **(A, B)** represents levels of estrogen and androgen respectively. N = 20 per group. *P < 0.05; ***P < 0.001 versus control group.

Upon exposure to GEN, the serum estrogen levels of the mice were significantly increased at the dose of 5 mg/kg/day (*P* < 0.05), 50 mg/kg/day (*P* < 0.05) and 250 mg/kg/day (*P* < 0.001), while there was no obvious difference between 0.5 mg/kg/day and the control (Figure 3A). However, we didn't find any statistically significant difference in androgen while the level might be slightly lower than the control at the dose of 50 mg/kg/day and 250 mg/kg/day (Figure 3B).

### Metabolism analysis

A total of 163 kinds of metabolites were identified in mice testis from control and 5 mg/kg/day. Of these metabolites, eight (Dehydroepiandrosterone, Dodecanoic acid, Capric acid, Trizma acetate, 7b-Hydroxycholesterol, Dodecanedioic acid, Pyroglutamic acid, and Xanthurenic acid) were aberrantly changed in 5 mg/kg/day group (Supplementary Table 1). And dehydroepiandrosterone was the most significantly changed metabolite (*P* = 0.011).

### Changes of relative expression levels of miR-17-92 cluster in mice testis

**Figure 4:**
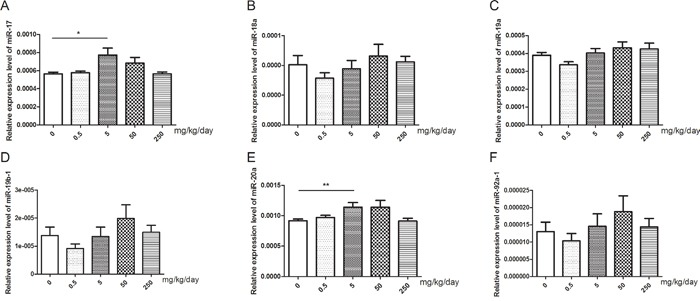
Changes of miR-17-92 expression in mice testis after treated with GEN at different doses **(A-F)** represents relative expression level of miR-17, miR-18a, miR-19a, miR-19b-1, miR-20a, and miR-92a-1, respectively. N = 20 per group. *P < 0.05; **P < 0.01 versus control group.

After treatment with GEN, we found that the relative expression levels of miR-17 and miR-20a were significantly increased at the dose of 5 mg/kg/day (Figure 4, *P* < 0.05, *P* < 0.01, respectively). But the relative expression levels of miR-18a, miR-19a, miR-19b-1, and miR-92a-1 did not show significantly changed after treatment with GEN (Figure 4).

### Target genes prediction and pathway enrichment of miR-20a

**Figure 5:**
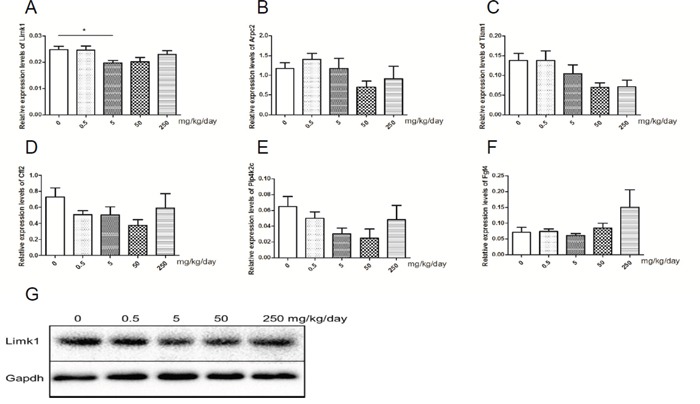
Relative expression levels of target genes of miR-20a **(A-F)** represents relative expression level of *Limk1*, *Arpc2*, *Tiam1*, *Cfl2*, *Pip4k2c* and *Fgf4* in mice testis, respectively. N = 20 per group. G represents levels of protein of LIMK1 in mice testis. *P < 0.05 versus control group.

miR-20a was the only miRNA that differentially expressed in human and mice samples. Therefore, we considered miR-20a as the key miRNA concerning abnormal spermatogenesis induced by GEN. Via the target genes prediction and pathway enrichment analysis of human and mice genes, we identified four pathways (regulation of actin cytoskeleton, axon guidance, pancreatic cancer and pathways in cancer) that exist in human and mice genes (Supplementary Figure 5A-5D). Of the four pathways, regulation of actin cytoskeleton was reported to involve in the microtubule-based processes of spermatogenesis [23]. In this pathway, six target genes (including *Limk1*, *Arpc2*, *Tiam1*, *Cfl2*, *Pip4k2c* and *Fgf4*) were both in human and mice (Supplementary Figure 5E). We tested these six target genes and found that only expression level of *Limk1* was significantly decreased in the group treated with 5 mg/kg/day GEN (Figure 5A, *P* < 0.05). The protein levels of LIMK1 showed the similar results (Figure 5G).

### Dual-luciferase report assay

**Figure 6:**
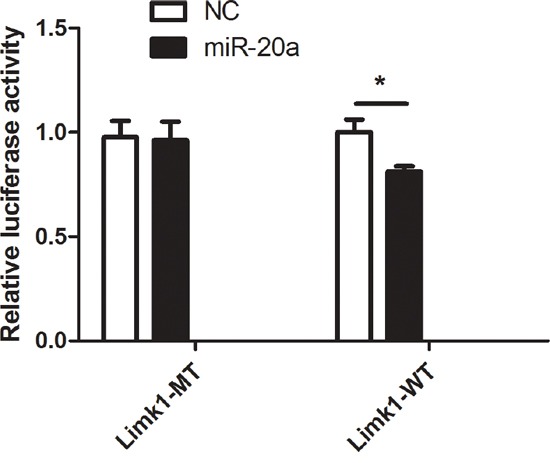
Dual-luciferase report assay The figure represents the dual-luciferase report assay result (**P < 0.01).

To investigate whether miR-20a directly bind to the 3’UTR regions of *Limk1*, we performed dual-luciferase report assay by constructing the mutant and wild type luciferase reporter plasmids containing the binding region of the 3’UTR of *Limk1* mRNA. We found that co-transfection of miR-20a mimics and pGL3-Limk1 3’UTR reporter plasmids significantly decreased the luciferase activity in 293T cells compared to the control (Figure 6, *P* < 0.01). This result suggested that miR-20a could directly target *Limk1*.

## DISCUSSION

In this study, we found that in infertile male subjects, sperm motility was lower in relative higher GEN dose group (Group3) while the relative expression levels of seminal plasma miR-19b-1, miR-20a and miR-92a-1 were higher in corresponding groups. These results suggested that miR-17-92 cluster might be involved in abnormal spermatogenesis induced by GEN in infertile subjects. Because all the subjects recruited were infertile and the urine exposure levels of GEN varied widely, we selected adult male ICR mice to determine whether miR-17-92 cluster plays roles in GEN induced abnormal spermatogenesis. Whether GEN could disrupt spermatogenesis remains controversial. It may depend on the dose, time, age and species. In 2000, Roberts et al., [24] found that gestational plus lactational exposure to GEN and subsequent dietary exposure to GEN had no adverse effects on gametogenic function in rats. However, in 2005, Svechnikov et al., [25] examined the effects of long-term dietary administration of GEN (21.1 mg/animal per day) on rats and found that GEN could influence spermatogenesis and significantly inhibited Leydig cell steroidogenesis. In 2014, Jones et al., [26] demonstrated that simultaneous gestational exposure to GEN and DEHP induced long-term alterations in testis development and function. Besides, GEN has also been reported to mitigate radiation-induced testicular damage in male C3H/HeN mice with 200 mg/kg body weight administration [27] as well as recover spermatogenesis in the busulfan-treated rat testis orally daily at a dose of either 50 or 100 mg/kg [28]. In our present study, we found that ICR mice orally at a dose of 5 mg/kg/day for 35 days could induce abnormal spermatogenesis to some extent, mainly on mice VAP, VSL and VCL. All these results indicate that the reproductive toxicity of GEN is not simple linely dose-response. Further researches are needed to determine the exact dose-response correlations.

GEN could mimic structure of oestradiol and bind both estrogen receptors [5], so we studied the serum sex hormone levels of the treated mice. It showed that serum estrogen levels of those mice were significantly increased at the dose of 5 mg/kg/day, 50 mg/kg/day and 250 mg/kg/day. However, only at 5 mg/kg/day, we found slight abnormal spermatogenesis. Thus, we carried out metabolism analysis and found that eight metabolites were aberrantly changed in 5 mg/kg/day group, while five of them (Dehydroepiandrosterone, Capric acid, Trizma acetate, Dodecanedioic acid, and Pyroglutamic acid) were only differently changed in 5 mg/kg/day group. These prompted us that underlying mechanism involved slight abnormal spermatogenesis induced by GEN rather than mimic estrogen. It was reported that miRNAs were concerned about spermatogenesis [29] as well as miR-17-92 cluster [21]. According to present study, we detected the relative expression levels of miR-17-92 both in male seminal plasma and mice testis. We found that miR-20a was the only miRNA of miR-17-92 cluster differentially expressed both in human and mice samples. These findings suggested that miR-20a might be one of the key miRNAs that is involved in abnormal spermatogenesis induced by GEN.

As miRNAs play their roles by regulating target genes, we identified target genes of miR-20a both in human and mice by bioinformatic methods. After analyzing the pathway enrichment of the target genes, we found that four pathways were both enriched in human and mice. In the four pathways, regulation of actin cytoskeleton was reported to be involved in spermatogenesis through microtubules [23]. Finally, we found that relative expression level of *Limk1* was significantly decreased in mice testis treated with GEN at 5mg/kg/day. The result of 3’ UTR luciferase assay showed that *Limk1* was targeted by miR-20a. The stability of actin cytoskeletal structures plays a central role in regulating cell motility and morphogenesis [30]. It has been showed that both LIMK1 and LIMK2 regulate actin cytoskeletal reorganization by the Rho family GTPases. Besides, RhoB/ROCK/LIMK1 pathway plays a crucial role in the regulation of Sertoli-germ cell adherens junction dynamics [31]. These studies suggested that abnormal LIMK1 expression may disrupt the function of actin cytoskeletal and RhoB/ROCK/LIMK1 pathway, thus it may affect cell motility and Sertoli-germ cell adherens junction dynamics. In anaplastic thyroid cancer, miR-20a could inhibit cellular proliferation and cell invasion via decreasing LIMK1 protein expression [32]. Furthermore, in cutaneous squamous cell carcinoma, researchers also found that miR-20a could inhibit A431 and SCL-1 proliferation and metastasis, and LIMK1 was a direct target gene of miR-20a [33]. Besides, is has been reported that GEN could down-regulate some important proteins for actin cytoskeleton [34], and *Limk1* is a key regulator for actin cytoskeleton, thus we hypothesized that *Limk1* may also be regulated by GEN. In present study, we found *Limk1* was significantly suppressed in GEN treated group at dose of 5 mg/kg/day, and VAP, VSL and VCL were significantly decreased in corresponding group. This phenomenon may explain that lower expression levels of Limk1 inhibited the actin cytoskeletal reorganization, and this process induces lower cell motility. However, no histopathological effect was found in the testis. Because of all the ICR mice were adult, their testis have been fully developed, and the exposure time may be not enough to influence testicular histopathology.

In summary, our findings demonstrated that GEN could induce slight abnormal spermatogenesis both in human and mice. miR-20a might be a key inhibitor in this process via targeting *Limk1* to lower cell motility.

## MATERIALS AND METHODS

### Subjects

Subjects were recruited from affiliated hospitals of Nanjing Medical University between March 2004 and June 2008 (NJMU Infertility Study). This study was approved by the Institutional Ethics Committee of Nanjing Medical University. All activities involving subjects were under full compliance with government policies and the Helsinki Declaration. All the 130 infertile subjects were the male partners of couples who attended affiliated hospitals of Nanjing Medical University because of their inability to conceive for at least 12 months. All participants provided informed consent and completed a questionnaire including information about age, height, weight, smoking and drinking status and other lifestyle factors.

### Urinary GEN measurement

Urinary concentration of GEN was analyzed by a sensitive LC-MS/MS method (Waters 2695 and Water Quattro Premier, USA). The detail sample pretreatment methods have been described previously [6]. The samples were analyzed by ultra-high performance liquid chromatography-tandem mass spectrometry (UPLC-MS/MS). The limit of detection for GEN was 0.04 ng/ml. Creatinine (CR) concentrations were used to adjust for variable urine dilution in spot samples. And the urinary CR concentrations were measured by colorimetric assay with an automated chemistry analyzer (7020 Hitachi, Japan). Quality control samples were analyzed in each analytical series in parallel with unknown samples.

### Chemicals

GEN (CAS no. 446-72-0) was obtained from Nanjing Spring & Autumn Biological Engineering (Nanjing, China). Corn oil was purchased from Aladdin (Shanghai, China). All chemicals were analytically graded (HPLC > 98%).

### Animal treatment

Seven-week-old ICR mice were purchased from the SLAC Laboratory Animal (Shanghai, China), and were housed in an animal care facility free with specific pathogen with 12:12-hour-dark cycle. They were fed a soy-free animal diet and used after 1 week of quarantine and acclimatization. The protocol used in this study was approved by Animal Care and Use Committee at Nanjing Medical University, and the animals were maintained according to the Guidelines for Animal Experiments at Nanjing Medical University. Body weight was measured daily during this study. Mice were randomly divided into five groups (n = 20 for each group), including a vehicle group and four GEN treated groups. The duration time of treatment in present study was selected according to the timing of mouse spermatogenesis [35]. The vehicle group mice received corn oil orally for 35 consecutive days while the treated group mice received orally corn oil mixed with GEN (GEN concentrations were 0.5, 5, 50 and 250 mg/kg/day for each treated group). The selection of doses were refer to the daily intake of GEN in human [36] and previous studies about GEN on mice models [37, 38]. One day after the last administration, all mice were sacrificed by cervical dislocation. The testicles of each mouse were dissected out and weighed. Left testis was selected for gene expression and western blotting, and right testis was selected for histology. The right epididymis was selected to analyze the sperm quality.

### Histology

The right testis of each mouse was embedded in paraffin, sectioned (5 μm) and stained with haematoxylin and eosin (H&E) for histology.

### Analysis of sperm quality in epididymal spermatozoa of mice

The right epididymis for each mouse was dissected out for the analysis of sperm quality. Eleven parameters of mice sperm including sperm concentration (×10^9^/ml), sperm motility (%), progressive (%), VAP (μm/s), VSL (μm/s), VCL (μm/s), ALH (μm), BCF (Hz), STR (%), LIN (%) and sperm activity (including rapid, medium, slow and static, %) were measured by IVOS sperm Analyzer (Hamilton Thorne Biosciences, USA). All analysis was run in a blinded fashion by one colleague.

### Analysis of serum sex hormone levels

The levels of estrogen and androgen of blood serum from each mouse were measured by using the mouse estrogen and androgen ELISA kit (Enzyme-linked Biotechnology, Shanghai, China).

### Metabolism analysis

Fifty milligram testis of mice were mixed with 750 μl ultra-pure water and then ultra-sonicated for 5 min (power, 70%). After this, 150 μl supernatant were gently mixed with the isotope labeled internal standard solution containing CR, L-valine, Maleic-acid, Nicotinic-acid, Thymine, Pentanedioic-acid, L-phenyl-alanine, N-(4-hydroxyphenyl), N-benzoyl-glycine, Indole-3-ACETIC-acid, Estrone, Progesterone, Pentadecanoic-acid, Tetracosnoic-acid. Then 450 μl methyl alcohol was added to precipitated protein, after 30 seconds vortex and 20000g centrifuging for 15 minutes, the supernatant was extracted into 1.5 ml centrifuge tube. The target analytes were concentrated under a speed vacuum concentrator at room temperature, and reconstituted in 10 ml ultrapure water, then injected into a Q-exactive UPLC-MS/MS system for analysis.

### RNA isolation and cDNA synthesis

Total RNAs including miRNAs in human seminal plasma were isolated using miRURY RNA Isolation Kit-Biofluids (Exiqon, Vedbeak, Denmark) following the manufacturer's instructions. TRIzol reagent (Invitrogen, USA) was chosen to isolate total RNAs from mice testis. The synthesis of cDNA was performed with 500 ng RNA using PrimeScript™ RT reagent Kit (Perfect Real Time) (Takara, Tokyo, Japan) according to the manufacturer's instructions.

### Real-time quantitative PCR analysis

ABI 7900 was used to determine the relative expression levels of selected miRNAs by Real-Time PCR. U6 was chosen as reference. Real-time PCR was repeated three times for every sample. The relative expression level was represented as 2^(-ΔCt)^, The primers were listed in Supplementary Table 2.

### Target genes prediction and pathway enrichment

TargetScan 7.0 (http://www.targetscan.org) was used for target genes prediction and DAVID 6.7 (https://david.ncifcrf.gov) was selected for pathway enrichment in human and mouse. Venn diagram was performed by website tools (http://bioinfogp.cnb.csic.es/tools/venny/).

### Dual-luciferase report assay

Human 293T cells were purchased from Chinese academy of sciences (Shanghai, China) and seeded on 24-well plate 24h and cultured to 50% before transfection. The transfection was carried out by Lipofectamine 2000 (Invitrogen Corp, CA, US). Cells were transfected with 50 nM miR-20a mimics, negative control (NC), 500 ng pGL3-Limk1-miR-20a-WT and pGL3-Limk1-miR-20a-Mut on 24-well plates respectively. Three nanogram of pRL-SV40 (a Renilla luciferase vector) was also co-transfected to normalize the differences in transfection efficiency. After 24h of transfection, the luciferase activities were performed with a dual-luciferase reporter system (Promega, Madison, WI) according to the manufacturer's instructions.

### Western blot analysis

The total mice testicular proteins were extracted and then quantified with a BCA protein assay kit. LIMK1 protein levels were determined by Western blot using a recombinant rabbit monoclonal anti-Limk1 antibody (Abcam, Kendall square, MA, USA, 1: 1000 dilution). GAPDH protein was detected by a mouse monoclonal anti-GAPDH antibody (Beyotime, Jiangsu, China, 1: 1000 dilution). For analysis, the bands on the blots were measured by image analysis software (Stratagene, La Jolla, CA).

### Statistical analysis

All values were shown as mean ± standard error (S.E.). All dates were analyzed using PASW statistics version 18.0 (SPSS, Chicago, USA). Statistical significance of multiple treatments was determined by one-way analysis of variance (ANOVA) followed by the Dunnett's test when appropriate. *P* < 0.05 was considered statistically significant.

## SUPPLEMENTARY MATERIALS FIGURES AND TABLES


