# Supplementary material for: Genistein up-regulates miR-20a to disrupt spermatogenesis via targeting Limk1
Source: Oncotarget. 2017 May 5;8(35):58728–37. doi: 10.18632/oncotarget.17637 (PMC5601687; doi:10.18632/oncotarget.17637)
Supplement: Supplementary file 1 [file oncotarget-08-58728-s001.pdf]

Genistein up-regulates miR-20a to disrupt spermatogenesis via targeting Limk1

SUPPLEMENTARY MATERIALS

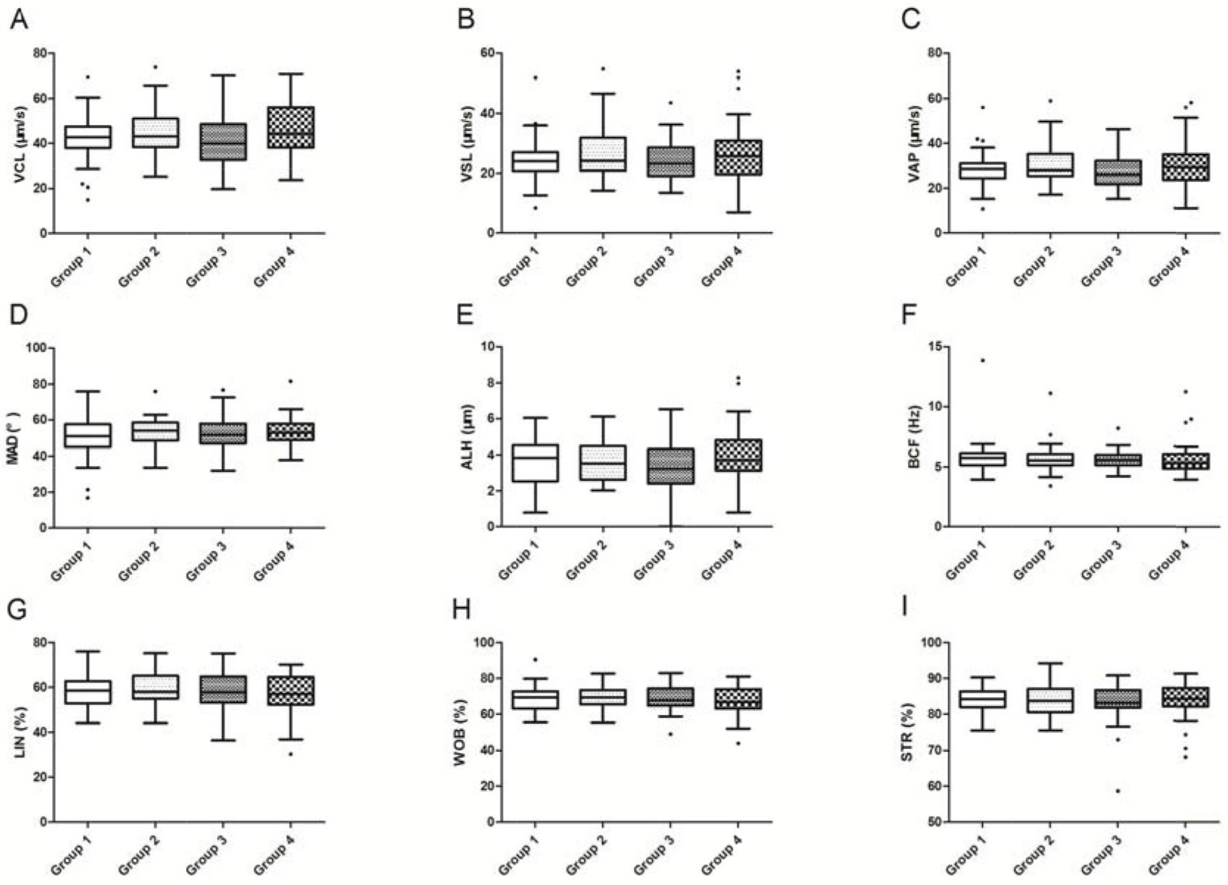

Supplementary Figure 1: Relationship between GEN exposure and other sperm characteristics in recruited subjects. (A-I) represents VCL, VSL, VAP, MAD, ALH, BCF, LIN, WOB and STP, respectively.

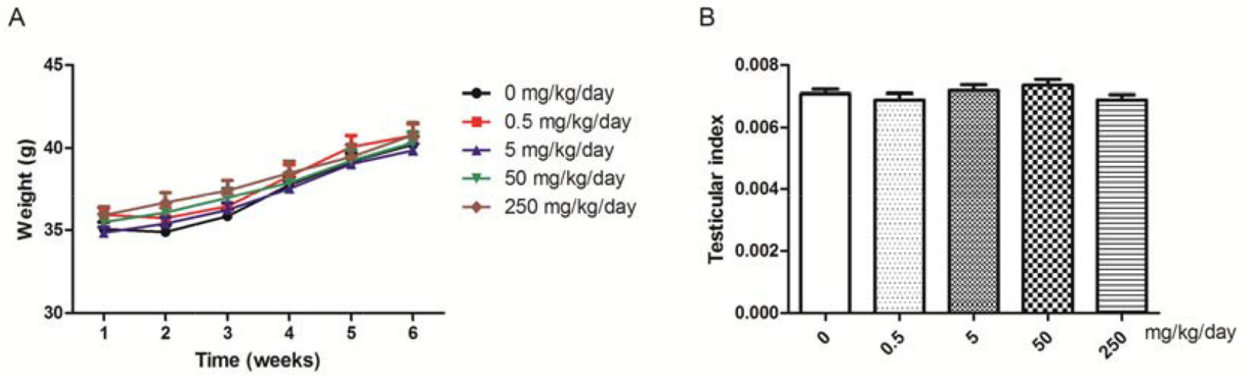

Supplementary Figure 2: Changes in body weight and testicular index of the mice after treated with GEN at different doses. **(A)** represents the changes of body weight weekly and **(B)** represents testicular index in different groups. N = 20 per group.

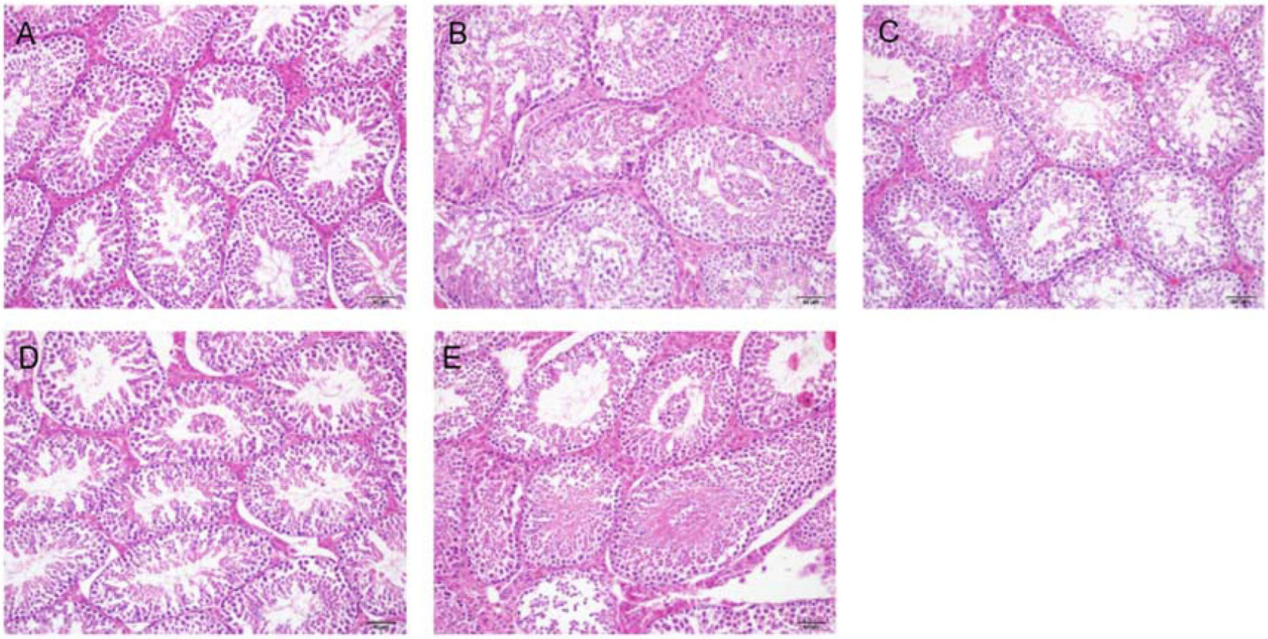

Supplementary Figure 3: Microscopy of cross section of H&E stained testis from control and GEN treated mice. (A-E) represents testis from mice treated with GEN at doses of 0, 0.5, 5, 50, 250 mg/kg/day, respectively. Scale bars = 50µm. N = 6 per group.

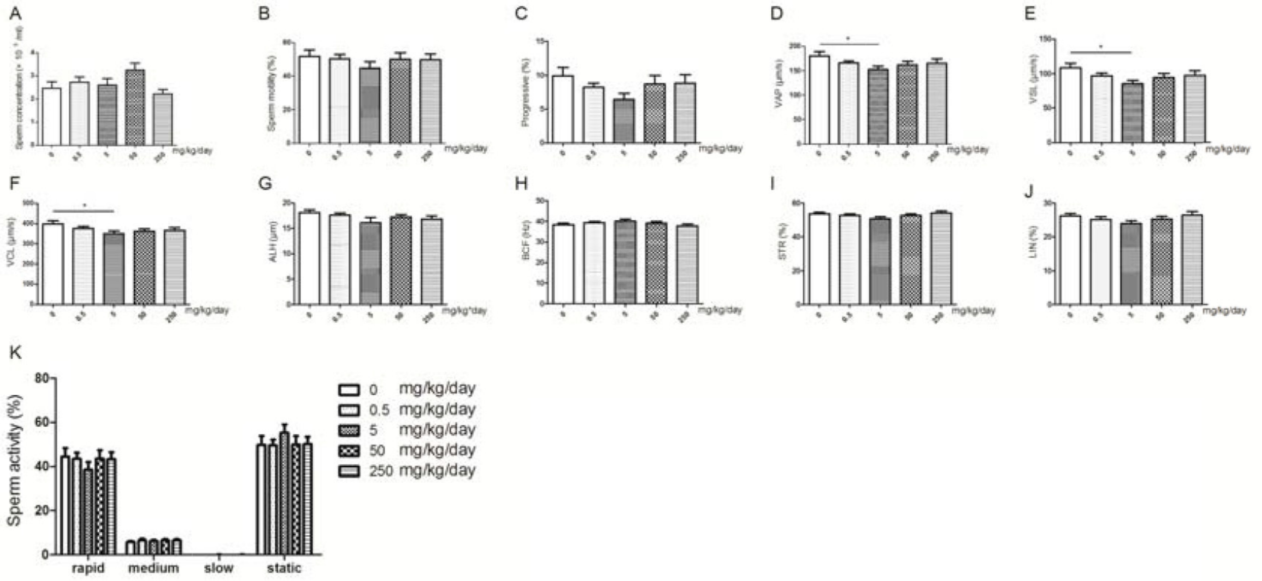

Supplementary Figure 4: Changes in sperm quality of mice after treated with GEN at different doses. **(A-K)** represents sperm concentration, sperm motility, progressive, VAP, VSL, VCL, ALH, BCF, STR, LIN & sperm activity, respectively. N = 20 per group. **P* < 0.05 versus control group.

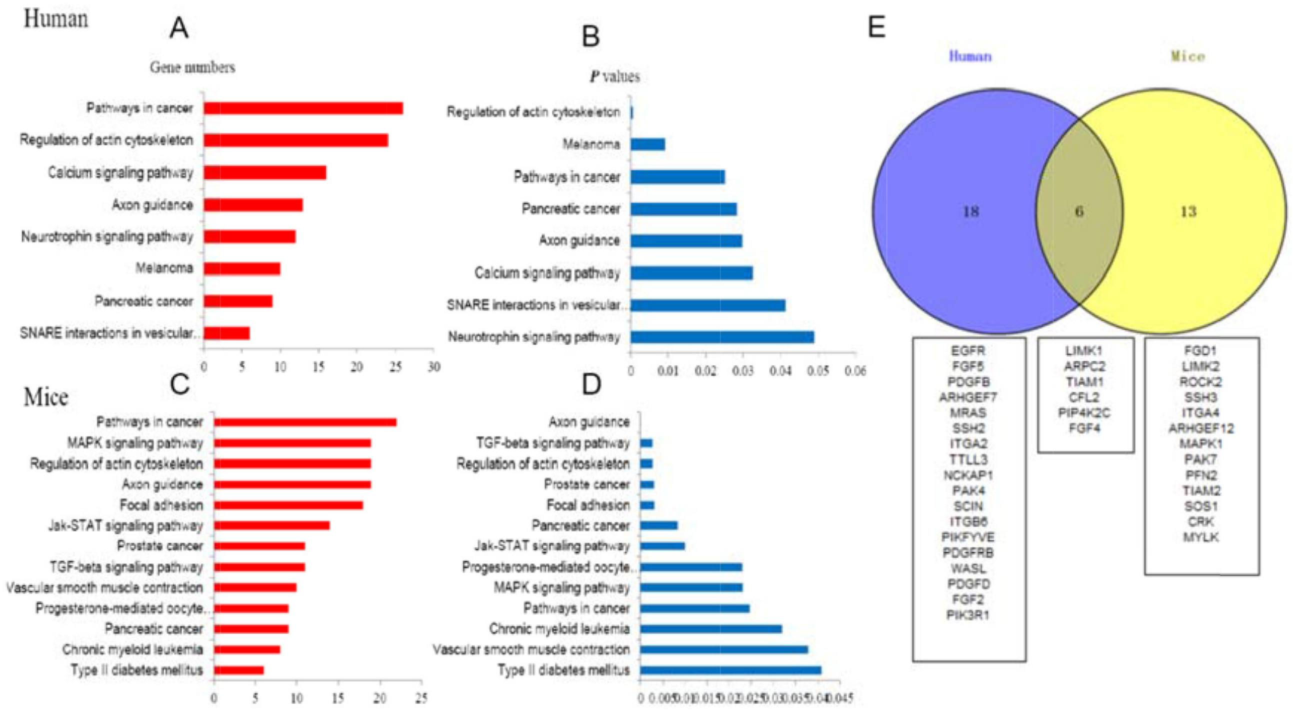

Supplementary Figure 5: Target genes prediction and pathway enrichment of miR-20a in human and mice. (A and C) represent numbers of target gene of miR-20a enriched in pathways in human and mice respectively; **(B and D)** represent *P* value for pathway enrichment respectively. **(E)** represents the target genes both in human and mice by Venn diagram; green (a) and blue (b) spot represents specific target genes enriched in regulation of actin cytoskeleton pathways in human and mice, respectively; red (c) spot represents target genes both enriched in regulation of actin cytoskeleton pathways in human and mice.

Supplementary Table 1: Metabolism results

Metabolite name	Fold vs Control	*P* value
Dehydroepiandrosterone	0.336	0.011
Dodecanoic acid	1.435	0.020
Capric acid	1.419	0.021
Trizma Acetate	0.695	0.031
7b-Hydroxycholesterol	0.576	0.033
Dodecanedioic acid	0.654	0.038
Pyroglutamic acid	0.754	0.043
Xanthurenic acid	0.444	0.043

Supplementary Table 2: Primers for miR-17-92 and Limk1, as well as references

Name	Primer-F (5'-3')	Primer-R (5'-3')
hsa-miR-17	ACACTCCAGCTGGGCAAAGTGCTTACAGTGC	CTCAACTGGTGTCGTGGAGTCGGCAATTCAGTTGAGCTACCTGC
hsa-miR-18a	ACACTCCAGCTGGGACTGCCCTAAGTGCTCC	CTCAACTGGTGTCGTGGAGTCGGCAATTCAGTTGAGCCAGAAGG
hsa-miR-19a	ACACTCCAGCTGGGTGTGCAAATCTATGCAA	CTCAACTGGTGTCGTGGAGTCGGCAATTCAGTTGAGTCAGTTTT
hsa-miR-19b-1	ACACTCCAGCTGGGAGTTTTGCAGGTTTGCA	CTCAACTGGTGTCGTGGAGTCGGCAATTCAGTTGAGGCTGGATG
hsa-miR-20a	ACACTCCAGCTGGGTAAAGTGCTTATAGTGC	CTCAACTGGTGTCGTGGAGTCGGCAATTCAGTTGAGCTACCTGC
hsa-miR-92a-1	ACACTCCAGCTGGGAGGTTGGGATCGGTTGC	CTCAACTGGTGTCGTGGAGTCGGCAATTCAGTTGAGAGCATTGC
mmu-miR-17	ACACTCCAGCTGGGCAAAGTGCTTACAGTGC	CTCAACTGGTGTCGTGGAGTCGGCAATTCAGTTGAGCTACCTGC
mmu-miR-18a	ACACTCCAGCTGGGACTGCCCTAAGTGCTC	CTCAACTGGTGTCGTGGAGTCGGCAATTCAGTTGAGCAGAAGGA
mmu-miR-19a	ACACTCCAGCTGGGTGTGCAAATCTATGCAA	CTCAACTGGTGTCGTGGAGTCGGCAATTCAGTTGAGTCAGTTTT
mmu-miR-19b-1	ACACTCCAGCTGGGAGTTTTGCAGGTTTGCA	CTCAACTGGTGTCGTGGAGTCGGCAATTCAGTTGAGGCTGGATG
mmu-miR-20a	ACACTCCAGCTGGGTAAAGTGCTTATAGTGC	CTCAACTGGTGTCGTGGAGTCGGCAATTCAGTTGAGCTACCTGC
mmu-miR-92a-1	ACACTCCAGCTGGGAGGTTGGGATTTGTCGC	CTCAACTGGTGTCGTGGAGTCGGCAATTCAGTTGAGAGCATTGC
U6	CTCGCTTCGGCAGCACA	AACGCTTCACGAATTTGCGT
Limk1	GGGGACACCTACACACTGGT	GGATCTGTTCGATGACTGGAGT
Gapdh	GCACCGTCAAGGCTGAGAAC	GGATCTCGCTCCTGGAAGATG
URP	TGGTGTCGTGGAGTCG	
